# Joint Influence of Recreational Physical Activity and Diet Quality on MAFLD: A National Study of Dose–Response and Synergy

**DOI:** 10.1002/hsr2.72250

**Published:** 2026-03-30

**Authors:** Genzhong Xu, Ming Luo

**Affiliations:** ^1^ Department of Spine Surgery and Musculoskeletal Tumor Zhongnan Hospital of Wuhan University Wuhan China

**Keywords:** dietary quality, metabolic dysfunction‐associated fatty liver, NHANES, physical activity

## Abstract

**Background and Aims:**

Utilizing data from the 2017–2020 National Health and Nutrition Examination Survey (NHANES), this study examined the dose–response relationships and potential synergistic effects of physical activity (PA) and dietary quality (DQ) on the risk of metabolic dysfunction‐associated fatty liver disease (MAFLD) among US adults.

**Methods:**

A nationally representative sample of 6511 participants was analyzed. PA was quantified as weekly recreational physical activity time, and DQ was assessed using the Healthy Eating Index–2015. Weighted multivariable logistic regression models, adjusted for sociodemographic, metabolic, and behavioral confounders, were used to evaluate associations. Dose–response patterns were examined using smooth curve fitting and segmented regression.

**Results:**

Both higher PA and DQ were independently associated with reduced MAFLD risk (DQ: OR = 0.984, 95% CI = 0.981–0.987; PA: OR = 0.974, 95% CI = 0.969–0.978; both *p* < 0.0001), with distinct patterns. For PA, each 1‐h increase up to 6.776 h/week reduced risk by 9.2% (95% CI: 7.8–10.7%; *p* < 0.001), with no additional benefit beyond this threshold (*p* = 0.818). For DQ, each 1‐point increase in the Healthy Eating Index–2015 score reduced risk by 0.9% for scores ≤ 50 and by 2.2% for scores > 50. Compared to low PA (≤ 2.5 h/week), moderate (2.5–6.677 h/week) and high PA (> 6.677 h/week) were associated with a 34.1% and 47.7% lower risk, respectively. Moderate and high DQ reduced risk by 15.2% and 38.0% versus low DQ. The combined high‐PA/high‐DQ group showed the greatest risk reduction (OR = 0.325, 95% CI: 0.278–0.381). In individuals with low DQ, increasing PA from low to high reduced risk by 35.2%–39.3%, whereas improving DQ at low PA levels yielded a more modest reduction (13.4%–29.8%).

**Conclusions:**

PA and DQ were independently associated with a lower MAFLD risk, with PA exhibiting a threshold effect (optimal at ~400 min/week) and DQ demonstrating cumulative benefits at higher scores. Combining high PA and high DQ yields the strongest protective effect, underscoring the importance of integrated lifestyle interventions for MAFLD prevention.

## 1 Introduction

Metabolic dysfunction‐associated fatty liver disease (MAFLD) now affects roughly one in three US adults, reflecting its emergence as a worldwide public‐health challenge [1, 2]. The condition clusters with metabolic syndrome and is defined by hepatic fat accumulation alongside heightened cardiometabolic risk [3]. Its development stems from a complex interplay of excess adiposity, impaired insulin action, and atherogenic lipid profiles. Among the modifiable drivers, physical‐activity patterns and overall dietary quality are increasingly recognized as key, yet still insufficiently studied, influences on disease trajectory [4, 5].

While prior investigations have established general associations between lifestyle factors and liver disease risk, several critical gaps remain [6, 7, 8]. First, existing studies predominantly relied on the older non‐alcoholic fatty liver disease (NAFLD) definition rather than the updated MAFLD criteria that better capture metabolic dysfunction [4, 5]. Second, the nonlinear relationships and optimal thresholds for PA duration and DQ for MAFLD prevention remain undefined—knowledge essential for actionable public health guidance [9]. Third, prior research on combined PA and DQ effects has typically used binary categorizations, limiting understanding of dose–response interactions [10]. These methodological limitations underscore the need for more rigorous, objectively assessed investigations that can precisely characterize dose‐response relationships and synergistic effects.

To address these gaps, we analyzed data from the 2017–2020 cycles of the National Health and Nutrition Examination Survey (NHANES) with three primary objectives: (1) to examine the independent associations of PA and DQ with MAFLD risk; (2) to model their nonlinear dose‐response patterns using segmented regression to identify precise thresholds; and (3) to quantify the joint influence of PA and DQ through comprehensive nine‐group cross‐classification based on the derived threshold values.

## 2 Materials and Methods

### 2.1 Study Participants and Data Collection

Data from the NHANES—A cross‐sectional survey conducted by the Centers for Disease Control and Prevention's National Center for Health Statistics to assess the prevalence of diseases and their risk factors in the US population—were utilized in this study. The inclusion criteria were as follows: (1) completion of vibration‐controlled transient elastography; (2) complete physical activity records; (3) availability of dietary information. We excluded participants who lacked sufficient clinical data to confirm a MAFLD diagnosis. The National Center for Health Statistics Research Ethics Review Board approved the study protocol (no. 2018‐01), and all individuals provided written informed consent.

### 2.2 Diagnosis of MAFLD

MAFLD was diagnosed according to established international criteria, requiring evidence of hepatic steatosis in addition to at least one of the following: overweight/obesity, type 2 diabetes mellitus, or evidence of metabolic dysfunction [1]. Overweight/obesity was characterized by a body mass index (BMI) of ≥ 25 kg/m². Metabolic dysfunction was defined as the presence of two or more of the following components: (1) elevated waist circumference (> 102 cm in men or > 88 cm in women); (2) hypertension, defined as systolic/diastolic blood pressure ≥ 130/85 mmHg or current use of antihypertensive medication; (3) hypertriglyceridemia (triglycerides > 1.70 mmol/L) or documented use of lipid‐lowering therapy; (4) reduced high‐density lipoprotein cholesterol (< 1.0 mmol/L in men or < 1.3 mmol/L in women); (5) prediabetes; or (6) elevated high‐sensitivity C‐reactive protein (> 2 mg/L).

Hepatic steatosis was assessed non‐invasively using vibration‐controlled transient elastography with the controlled attenuation parameter. The values range from 100 to 400 dB/m, with higher values indicating greater hepatic fat content. Based on previously validated thresholds, steatosis severity was categorized into grades S0–S3, with CAP cut‐offs of 248, 268, and 280 dB/m for S1, S2, and S3, respectively [11]. In this study, hepatic steatosis was defined as a CAP value ≥ 268 dB/m, corresponding to at least moderate steatosis (≥ S2).

### 2.3 Assessment of Physical Activity

The Global Physical Activity Questionnaire in NHANES survey assesses the amount of time spent sitting and participating in typical physical activities during the past week. We used recreational physical activity time (RPAT) as a proxy for weekly physical activity. According to the 2020 WHO Physical Activity Guidelines, 1 min of vigorous‐intensity activity can be converted into 2 min of moderate‐intensity activity [12]. The guidelines recommend that adults engage in at least 150 min of moderate‐intensity physical activity per week (or 75 min of vigorous‐intensity activity, or an equivalent combination), with greater benefits observed with more than 300 min. The total minutes per week of physical activity were calculated, accounting for intensity, as follows: time of moderate‐intensity physical activity + (2 × time of vigorous − intensity physical activity).

### 2.4 Assessment of Dietary Quality

The dietary quality is evaluated based on the 2015 Healthy Eating Index (HEI‐2015) recommended by the United States Department of Agriculture [13]. NHANES provides two rounds of 24‐h dietary review data: the first round is completed face‐to‐face at the Mobile Medical Examination Center, and the second round is inquired by phone after 3–10 days. Dietary intake data were collected from two 24‐h recall interviews in NHANES and used to determine dietary scores from the food patterns equivalents database [14].

### 2.5 Description and Analysis of Covariates

In our research analysis, the demographic characteristics presented as potential confounding factors on the NHANES website were considered as covariates, including age, gender (male, female), race/ethnicity (Mexican Americans, other Hispanics, non‐Hispanic whites, non‐Hispanic blacks, other races), smoking at least 100 cigarettes (yes or no), alcohol use, sedentary activity/day, diabetes, obesity, and metabolic disorders. Alcohol use was categorized as follows: heavy drinking: > 1 time/week or > 6 cups/time; moderate drinking: 1–4 times/month or 3–6 cups/time; low drinking: < 1 time/month or 1–2 cups/time. Diabetes mellitus was defined by combining the following criteria: diabetes questionnaire responses, fasting blood glucose levels ≥ 7 mmol/L, and glycated hemoglobin levels ≥ 6.5%.

### 2.6 Statistical Analysis

All analyses incorporated NHANES complex survey design weights to ensure nationally representative estimates. Continuous variables were summarized as weighted means ± standard errors and assessed using Student's *t*‐test, while categorical variables were reported as weighted proportions (%) and assessed using the Rao–Scott adjusted *χ* ^2^ test.

The smooth curve fitting with weighted generalized additive models was used to visualize exposure‐response curves. The segmentation effect (two‐piecewise logistic regression) was used to identify potential thresholds (knots determined by a recursive algorithm). Three hierarchically adjusted models were constructed: model 1, crude association (unadjusted); model 2, adjusted for sociodemographics (age, gender, race), behaviors (alcohol use, smoking status, sedentary time); model 3, further adjusted for metabolic confounders (diabetes, obesity, metabolic syndrome) and mutual adjustment for DQ and PA.

PA was classified into three levels: low (≤ 2.5 h/week), moderate (2.5–6.677 h/week), and high (> 6.677 h/week). DQ was classified into tertiles according to the distribution of the NHANES population (low: HEI‐2015 ≤ 43.96; moderate: 43.96–54.99; high: HEI‐2015 > 54.99). Weighted logistic regression models were used to estimate the odds ratios (ORs) and 95% confidence intervals (95% CIs) for the association between PA and DQ with the risk of MAFLD, utilizing the same three adjustment models as above.

Interaction analysis was conducted by generating nine combined lifestyle groups through cross‐classifying PA and DQ tertiles. Adjusted ORs with 95% CIs were estimated using logistic regression, with the low‐PA/low‐DQ group as the reference. Analyses for threshold detection were conducted using R 4.3 and Empower Stats 4.2. Statistical significance was defined as a two‐tailed *p* < 0.05.

## 3 Results

### 3.1 Baseline Characteristics of Participants

**Figure 1:**
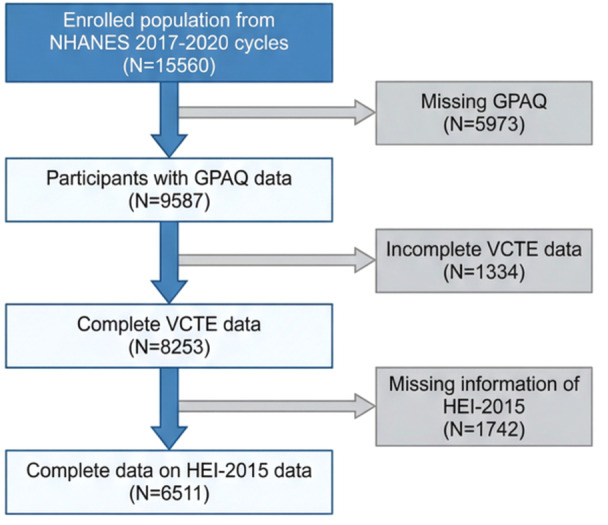
Flowchart of the selection in this study.

A total of 15,560 participants were initially included. After exclusions (5973 with missing data, 1334 with incomplete MAFLD criteria, and 1742 lacking HEI‐2015 assessments), 6511 participants (non‐MAFLD = 3575, MAFLD = 2936) remained in the final analysis (Figure 1). Participants in the MAFLD group were more likely to be older and male (*p* < 0.001). Furthermore, there was a greater prevalence of diabetes, metabolic dysfunction, and overweight/obesity in the MAFLD group (*p* < 0.001). Additionally, they had bigger waist circumferences, higher BMIs, and higher levels of FBG, triglycerides, and hemoglobin A1c (*p* < 0.001). Compared with the MAFLD group, participants in the non‐MAFLD group had more RPAT (5.16 ± 8.17 vs. 3.15 ± 6.11, *p* < 0.001), shorter sedentary time (5.72 ± 3.24 vs. 6.27 ± 3.46, *p* < 0.001), and improved dietary quality (51.09 ± 12.50 vs. 49.11 ± 11.61, *p *< 0.001) (Table 1).

**Table 1:** Weighted characteristics of the study population.

	Non‐MAFLD	MAFLD	*p* value
*N*	3575 (54.91%)	2936 (45.59%)	
Age (years, mean ± SD)	44.66 ± 17.82	51.20 ± 16.04	< 0.0001
Gender (%)			< 0.0001
Male	43.83	53.97	
Female	56.17	46.03	
Race/ethnicity (%)			< 0.0001
Mexican American	5.87	10.66	
Other Hispanic	7.54	6.98	
Non‐Hispanic White	63.95	64.49	
Non‐Hispanic Black	13.46	9.48	
Non‐Hispanic Asian	5.46	4.10	
Other race—including multi‐racial	3.73	4.29	
Alcohol use (%)			0.0004
Low drinking	24.26	27.01	
Moderate drinking	53.83	48.89	
Heavy drinking	21.92	24.10	
Smoked at least 100 cigarettes (%)			< 0.0001
No	39.15	44.06	
Yes	60.85	55.94	
Over‐weight/obesity (%)			< 0.0001
No	47.50	3.58	
Yes	52.50	96.42	
Diabetes status (%)			< 0.0001
No	94.57	75.31	
Yes	5.43	24.69	
Metabolic syndrome (%)			< 0.0001
No	38.80	100.00	
Yes	61.20		
Waist (cm)	91.49 ± 13.44	111.39 ± 14.63	< 0.0001
Body mass index (%)	26.32 ± 5.49	33.96 ± 7.05	< 0.0001
FBG (mmol/L)	5.63 ± 1.02	6.63 ± 2.29	< 0.0001
HbA1c (%, mean ± SD)	5.63 ± 1.02	6.63 ± 2.29	< 0.0001
Median CAP (dB/m, mean ± SD)	217.98 ± 36.88	320.91 ± 37.90	< 0.0001
ALT (IU/L, mean ± SD)	19.23 ± 16.14	27.03 ± 18.66	< 0.0001
AST (IU/L, mean ± SD)	20.99 ± 13.32	22.97 ± 12.06	< 0.0001
Triglyceride (µmol/L, mean ± SD)	1.24 ± 0.74	1.97 ± 1.42	< 0.0001
Sedentary hours (h/day, mean ± SD)	5.72 ± 3.24	6.27 ± 3.46	< 0.0001
HEI‐2015 total scores	51.09 ± 12.50	49.11 ± 11.61	< 0.0001
Recreation physicalactivity time (h/week, mean ± SD)	5.16 ± 8.17	3.15 ± 6.11	< 0.0001

Abbreviations: ALT, alanine aminotransferase; AST, aspartate aminotransferase; CAP, controlled attenuation parameter; FBG, fasting blood glucose; HbA1c, glycated hemoglobin A1c.

### 3.2 Threshold Effects of PA on MAFLD Risk

**Figure 2:**
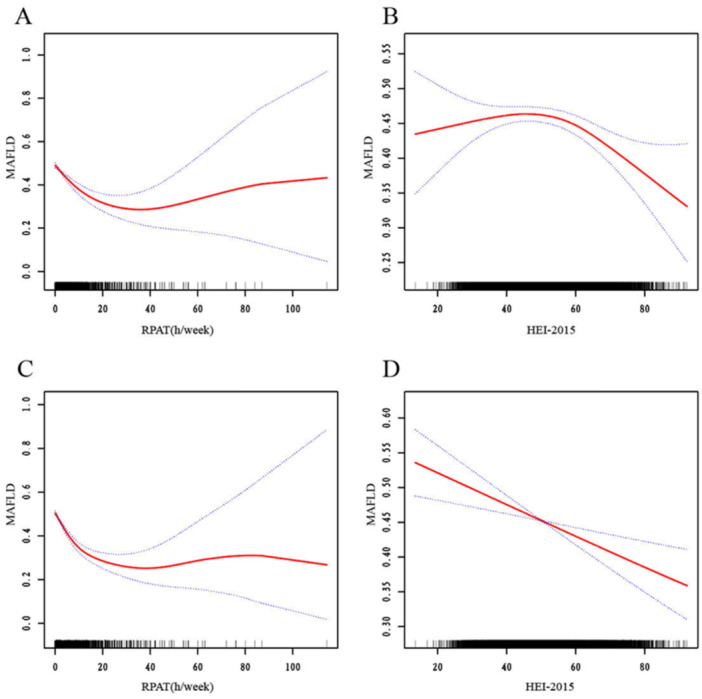
The smooth curve fitting for associations of HEI‐2015 total score and RPAT with MAFLD. (A, B) (unadjusted). (C, D) Adjusted for sociodemographics (age, gender, race), behaviors (alcohol use, smoking status, sedentary time), metabolic confounders (diabetes, obesity, metabolic syndrome), and mutual adjustment for RPAT/HEI‐2015.

Across all models (from unadjusted to fully adjusted), PA exhibited a consistent inverse association with MAFLD risk. In the fully adjusted model (model 3), each 1‐h/week increment in PA was associated with a 2.6% reduction in MAFLD risk (OR = 0.974, 95% CI: 0.969–0.978, *p* < 0.001). Nonlinear analyses revealed a threshold effect at 6.677 h/week. Below the threshold: Each 1‐h/week increase in PA (≤ 6.677 h/week) conferred a 9.2% risk reduction (OR = 0.908, 95% CI: 0.893–0.922, *p* < 0.001). Above the threshold, no further benefits were detected (OR = 1.001, 95% CI 0.993–1.009, *p *= 0.818) (Table 2). The threshold was statistically validated by a log‐likelihood ratio test (*p* < 0.001 for model fit improvement). Smooth curve fitting also demonstrated the same threshold effect, with the risk of MAFLD rapidly decreasing as PA increases and then gradually stabilizing, whether before adjustment or after adjustment (Figure 2).

**Table 2:** Threshold effect of the HEI‐2015 and RPAT with the risk of MAFLD.

	HEI‐2015 *β* (95% CI) *p* value	RPAT *β* (95% CI) *p* value
Linear model	0.984 (0.981, 0.987) < 0.0001	0.974 (0.969, 0.978) < 0.0001
Non‐linear model		
Inflection point	50.754	6.667
< Inflection point	0.991 (0.985, 0.997) 0.0048	0.908 (0.893, 0.922) < 0.0001
< Inflection point	0.978 (0.972, 0.984) < 0.0001	1.001 (0.993, 1.009) 0.8180
Log likelihood ratio	0.015	< 0.001

*Note:* Adjusted variables: sociodemographics (age, gender, race), behaviors (alcohol use, smoking status, sedentary time), metabolic confounders (diabetes, obesity, metabolic syndrome), and mutual adjustment for dietary quality (RPAT) and physical activity (HEI‐2015).

To align with international recommendations ( ≥ 2.5 h/week moderate activity) [6], participants were stratified into three categories: Low PA: ≤ 2.5 h/week (Reference) Moderate PA: 2.5–6.677 h/week High PA: > 6.677 h/week In fully adjusted models (model 3): moderate PA reduced MAFLD risk by 34.1% (OR = 0.659, 95% CI: 0.598–0.726, *p* < 0.001); High PA further reduced risk by 47.7% (OR = 0.523, 95% CI: 0.475–0.577, *p *< 0.001) (Table 3). While moderate PA significantly reduces the risk of MAFLD, the additional benefit of high PA is not statistically significant compared with moderate PA.

**Table 3:** Association of the HEI‐2015 and RPAT with the risk of MAFLD.

	Model 1 (OR (95%CI))	Model 2 (OR (OR (95%CI))	Model 3 (OR (95%CI))
RPAT (hour/week)			
Linear model	0.957 (0.952, 0.961)	0.962 (0.957, 0.967)	0.972 (0.967, 0.978)
Cutoffs			
Low	1.0	1.0	1.0
Middle	0.631 (0.582, 0.684)	0.642 (0.590, 0.698)	0.659 (0.598, 0.726)
High	0.430 (0.398, 0.466)	0.466 (0.428, 0.507)	0.523 (0.475, 0.577)
HEI‐2015			
Linear model	0.987 (0.984, 0.989)	0.979 (0.976, 0.981)	0.984 (0.981, 0.987)
Tertile			
Low	1.0	1.0	1.0
Middle	1.007 (0.936, 1.083)*	0.876 (0.811, 0.946)	0.848 (0.776, 0.927)
High	0.718 (0.667, 0.773)	0.571 (0.527, 0.619)	0.620 (0.565, 0.680)

Model 1: crude association (unadjusted).

Model 2: adjusted for sociodemographics (age, gender, race), behaviors (alcohol use, smoking status, sedentary time).

Model 3: further adjusted for metabolic confounders (diabetes, obesity, metabolic syndrome) and mutual adjustment for dietary quality (RPAT) and physical activity (HEI‐2015).

RPAT (hour/week): low: ≤ 2.5; middle: > 2.5, ≤ 6.66; high: > 6.667.

HEI‐2015: low ≤ 43.96; middle: > 43.96, ≤ 55.00; high > 55.00.

* *p* > 0.05.

### 3.3 Cumulative Benefits of Diet Quality on MAFLD Risk

Consistent across all models, higher DQ (as measured by HEI‐2015 total scores) was associated with progressively lower MAFLD risk. Each one‐point increase in HEI‐2015 total scores in the fully adjusted model (model 3) resulted in a 1.6% risk reduction (OR = 0.984, 95% CI: 0.981–0.987, *p *< 0.0001). Non‐linear analyses identified a gradient intensification effect at 50.8 points (Table 2): below 50.8, each one‐point increase reduced risk by 0.9% (OR = 0.991, 95% CI: 0.985–0.997, *p* = 0.0048); above 50.8, the protective effect doubled to 2.2% per point (OR = 0.978, 95% CI: 0.972–0.984, *p *< 0.0001), demonstrating cumulative benefits of sustained dietary improvements. Smooth curve fitting revealed that the low DQ group (HEI‐2015 < 50) did not show a protective effect before adjustment, but a protective effect emerged after adjustment (Figure 2). In comparison to the low DQ group, individuals with moderate DQ experienced a 15.2% reduction in MAFLD risk (OR = 0.848, 95% CI: 0.776–0.927, *p* < 0.001), while those with high DQ saw a 38.0% reduction in risk (OR = 0.620, 95% CI: 0.565–0.680, *p *< 0.001) (Table 3). Notably, while moderate DQ significantly lowered MAFLD risk, the additional protective effect of high DQ was statistically significant compared with moderate DQ.

### 3.4 Synergistic Effects of Combined Physical Activity and Dietary Quality

**Figure 3:**
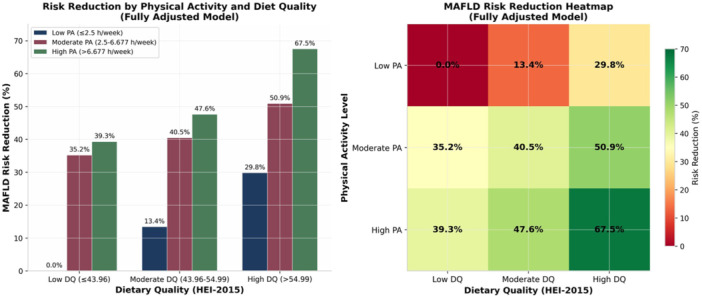
Synergistic effects of combined physical activity and dietary quality on MAFLD risk reduction. (A) Grouped bar chart showing percentage risk reduction across nine cross‐classified groups. (B) Heatmap visualization of risk reduction percentages, with darker green indicating greater protection. All values derived from the fully adjusted Model 3. Reference group: low PA/low DQ (0% reduction).

Cross‐classifying HEI‐2015 total scores tertiles and RPAT three categories revealed a dose‐dependent gradient in MAFLD risk reduction (Table 4, Figure 3): Figure 3 visualizes these interaction effects through both a grouped bar chart and a heatmap representation. The visualization clearly demonstrates that optimal protection was observed in the high‐HEI/high‐RPAT group (HH), which showed a 67.5% lower risk (OR = 0.325, 95% CI: 0.278–0.381, *p* < 0.0001) compared with the low‐HEI/low‐RPAT reference group (LL). The heatmap reveals a clear gradient pattern, with risk reduction increasing progressively from the top‐left (low PA/low DQ: 0%) to bottom‐right (high PA/high DQ: 67.5%) quadrant. High diet quality combined with moderate activity reduced risk by 50.9% (OR = 0.491, 95% CI: 0.418–0.577); moderate diet quality combined with high activity achieved a 47.6% reduction (OR = 0.524, 95% CI: 0.445–0.617) (both *p* < 0.0001).

**Table 4:** Synergistic Effect of the HEI‐2015 and RPAT with the risk of MAFLD.

			Model 1 (OR (95% CI))	Model 2 (OR (OR (95% CI))	Model 3 (OR (95% CI))
RPAT	HEI‐2015	*N*			
Low	Low	285	1.0	1.0	1.0
Middle	Low	365	0.701 (0.602, 0.816)	0.676 (0.577, 0.793)	0.648 (0.540, 0.778)
High	Low	1465	0.513 (0.445, 0.591)	0.586 (0.504, 0.681)	0.607 (0.510, 0.721)
Low	Middle	294	1.032 (0.942, 1.129)*	0.908 (0.826, 0.998)	0.866 (0.775, 0.967)
Middle	Middle	411	0.809 (0.704, 0.931)	0.709 (0.612, 0.820)	0.595 (0.506, 0.701)
High	Middle	1287	0.490 (0.428, 0.560)	0.478 (0.415, 0.551)	0.524 (0.445, 0.617)
Low	High	423	0.903 (0.821, 0.993)	0.705 (0.636, 0.780)	0.702 (0.623, 0.790)
Middle	High	461	0.467 (0.410, 0.531)	0.419 (0.366, 0.480)	0.491 (0.418, 0.577)
High	High	285	0.322 (0.282, 0.367)	0.288 (0.251, 0.331)	0.325 (0.278, 0.381)
*p* interaction			0.0003	0.0064	0.0360

Model 1: Crude association (unadjusted).

Model 2: Adjusted for sociodemographics (age, gender, race), behaviors (alcohol use, smoking status, sedentary time).

Model 3: Further adjusted for metabolic confounders (diabetes, obesity, metabolic syndrome) and mutual adjustment for dietary quality (RPAT) and physical activity (HEI‐2015).

RPAT (hour/week): low: ≤ 2.5; middle: > 2.5, ≤ 6.66; high: > 6.667.

HEI‐2015: low ≤ 43.96; middle: > 43.96, ≤ 55.00; high > 55.00.

* *p* > 0.05.

At low diet quality, increasing RPAT from low to high conferred a 35.2%–39.3% risk reduction (*p *< 0.0001), demonstrating activity‐driven protection even with poor diet. At low activity levels, improving diet from low to high yielded only a 29.8% risk reduction (OR = 0.702, 95% CI: 0.623–0.790), with non‐significant effects for moderate diet improvement (13.4%, 95% CI: 0.775–0.967) (Table 4). At moderate diet quality, increasing RPAT from low to high conferred a 40.5%–47.6% risk reduction (*p* < 0.0001). At moderate activity levels, improving diet from low to high yielded a 40.8%–50.9% risk reduction (OR = 0.702, 95% CI: 0.623–0.790).

The preventive effect of moderate PA was significant, while the effect of a moderate diet was not significant. Similarly, compared with moderate PA, the preventive effect of high PA did not significantly improve (further revealing the saturation effect of PA). Conversely, compared with a moderate diet, the effect of a high‐quality diet was significantly enhanced (further demonstrating the cumulative benefits of diet).

## 4 Discussion

Our analysis reveals three key phenomena: (1) a saturation effect for physical activity (optimal at 6.677 h/week or 400 min/week), (2) cumulative benefits of diet quality (HEI‐2015 > 50.8), and (3) synergistic risk reduction when combining both factors. Physical activity can have a significant preventive effect from the beginning, and its effect is most pronounced at 400 min per week. Continuing to increase weekly exercise time may not provide additional benefits. Conversely, a low‐quality diet did not show significant benefits, but the protective effect increased significantly when the HEI‐2015 total score exceeded 50.8 points.

Physical activity demonstrates a significant preventive effect against MAFLD, with the most pronounced benefits at 400 min per week. Notably, even moderate levels of physical activity (2.5–6.67 h/week) significantly reduced the risk of MAFLD by 34.1%, which in turn supports global recommendations that recommend moderate exercise (150 min/week) for a duration of 1 year [15]. This aligns with research in other conditions, such as a study showing no further reduction in all‐cause or cardiovascular mortality with more than 300 min/week of vigorous activity or 600 min/week of moderate activity [16]. Similarly, a dose–response analysis of physical activity and depression identified an optimal threshold at 7.175 h/week (1722 MET‐min/week) [17]. Kim et al. show that meeting PA Guidelines for leisure‐time PA (150 min/week) has beneficial effects on NAFLD, and 300 min of PA Guidelines had a lower risk for significant fibrosis or cirrhosis [8]. We speculate that when the duration of physical activity surpasses 400 min, the protective effect of physical activity against MAFLD is likely maximized.

The protective role of diet against MAFLD is well‐established, but the cumulative nature of this effect remains understudied [7, 9, 18]. Our study highlights that the dietary patterns, as measured by the HEI‐2015 score, exhibit a clear dose–response relationship with MAFLD risk. Dietary patterns, rather than individual nutrients, are more reflective of the complex interplay between foods and nutrients, which can lead to cumulative health effects [19]. High sodium intake might impair insulin signaling pathways, leading to dysregulation of insulin sensitivity and increased fat accumulation in the body [20]. On the other hand, specific nutrients present in dairy products, including milkfat, vitamin D, calcium, magnesium, potassium, and whey proteins, could potentially offer protective benefits against MAFLD [21].

When HEI‐2015 scores exceed 50, multiple favorable dietary factors work in synergy, significantly enhancing the preventive effect of diet on MAFLD. For example, a high HEI‐2015 score indicates a diet rich in fruits, vegetables, whole grains, and lean proteins, which collectively improve metabolic health through various mechanisms, such as reducing inflammation, including reducing inflammation, enhancing insulin sensitivity, and fostering a healthy gut microbiome [7, 10]. In contrast, a low HEI‐2015 score (indicating a suboptimal diet) may fail to provide these synergistic benefits, resulting in a less pronounced protective effect against MAFLD.

This study found that the combination of high levels of PA and high DQ (higher HEI‐2015 score) had the most significant effect on reducing the risk of MAFLD. Sports activities not only directly improve metabolic function but also enhance the metabolic benefits of a healthy diet [22]. For example, sports activities further promote the metabolic utilization of nutrients provided by a healthy diet by increasing energy expenditure and improving insulin sensitivity [23]. In addition, a healthy diet can provide necessary nutritional support for physical activity, reduce sports injuries, and promote recovery [24].

Previous research aligns with and extends the findings of the present study. The Korean Occupational Health Screening Project demonstrated that moderate‐to‐vigorous physical activity significantly reduces the incidence of NAFLD and promotes the regression of existing hepatic steatosis over a 5‐year follow‐up period [25]. In European populations, an analysis of 327,387 UK Biobank participants established that a composite healthy lifestyle‐encompassing diet, alcohol consumption, physical activity, sedentary behavior, sleep, and smoking is strongly associated with a lower risk of MAFLD [26]. Corroborating this, the Dongfeng–Tongji cohort study found that adherence to a similar multi‐factorial healthy lifestyle is linked to reduced MAFLD risk in an East Asian population [27]. However, some studies present nuanced perspectives. A randomized controlled trial of obese adults with NAFLD (*n* = 80) in the United States indicated that while combining intermittent fasting with exercise effectively reduces hepatic steatosis, this regimen offers no significant synergistic advantage over fasting alone [28]. Furthermore, a systematic review concordant with our conclusions emphasizes that effective management involves dietary strategies focused on caloric restriction and quality improvement, supported by regular moderate‐to‐vigorous exercise, typically 150–240 min of aerobic activity per week, for MAFLD prevention [29].

Based on the threshold effect of exercise and the cumulative effect of diet, we can draw a preliminary conclusion. Activity dominates in poor diets (39.3% risk reduction despite low HEI‐2015). Diet excels when activity is adequate (HH group achievements 67.5% reduction). This suggests physical activity establishes metabolic “priming” that enhances dietary responsiveness—a hepatic version of the “muscle‐liver crosstalk” observed in exercise‐nutrition studies [30, 31]. While both physical activity and diet are equally important, adherence to both can be challenging for most individuals. If dietary control is difficult, increasing weekly exercise can still provide significant benefits. Conversely, a well‐controlled diet without adequate exercise may not yield the highest preventive effect against MAFLD.

Even if the NHANES database used in this study made use of big sample data, there are still some drawbacks. Firstly, NHANES data are cross‐sectional and cannot determine causal links. Secondly, RPAT and dietary data are obtained through self‐report, which may result in recall bias. In addition, this study did not consider the impact of other potential lifestyle factors, such as sleep quality and stress levels, on MAFLD. These conclusions are still being supported by prospective cohort studies or randomized controlled trials, which are required for future studies.

## 5 Conclusion

This study demonstrates that both RPAT and dietary quality independently reduce the risk of MAFLD among US adults, with distinct dose‐response patterns. Specifically, RPAT shows a threshold effect, with significant risk reduction up to 6.776 h per week, while dietary quality exhibits a stronger protective effect when HEI‐2015 scores exceed 50. Combined high physical activity and high dietary quality confer the lowest MAFLD risk, highlighting the importance of integrating both lifestyle factors for optimal prevention. Notably, improving physical activity yields greater risk reduction in individuals with low dietary quality, whereas dietary improvements offer moderate benefits in those with low physical activity levels. These findings underscore the need for tailored public health strategies targeting both physical activity and dietary quality to mitigate MAFLD risk.

## Author Contributions

**Genzhong Xu:** data curation, formal analysis, visualization, validation, writing – original draft, and software. **Ming Luo:** conceptualization, validation, writing – review and editing, supervision.

## Ethics Statement

The National Center for Health Statistics Research Ethics Review Board approved the study protocol (ID: 2018‐01), and all individuals provided written informed consent.

## Conflicts of Interest

The authors declare no conflicts of interest.

## Transparency Statement

The corresponding author, Ming Luo, affirms that this manuscript is an honest, accurate, and transparent account of the study being reported; that no important aspects of the study have been omitted; and that any discrepancies from the study as planned (and, if relevant, registered) have been explained.

## Data Availability

The data that support the findings of this study are available from the corresponding author upon reasonable request.
